# Perioperative Pain Management in Patients Undergoing Total Hip Arthroplasty: Where Do We Currently Stand?

**DOI:** 10.7759/cureus.9049

**Published:** 2020-07-07

**Authors:** Georgios Tsinaslanidis, Prodromos Tsinaslanidis, Ravindra H Mahajan

**Affiliations:** 1 Trauma and Orthopaedics, George Eliot Hospital National Health Service Trust, Nuneaton, GBR; 2 Trauma and Orthopaedics, St. George's University Hospitals National Health Service Foundation Trust, London, GBR

**Keywords:** total hip replacement, total hip arthroplasty, pain management, preemptive analgesia, peri articular injection, type of anesthesia, acetaminophen

## Abstract

Total Hip replacement (THR) is a well-discussed topic, and it offers excellent results in patients suffering from end-stage osteoarthritis (OA). However, despite the fact that patients can fully bear weight immediately after the surgery, THR is often associated with a great amount of postoperative pain affecting recovery and rehabilitation. Therefore, the efficient management of pain is of paramount importance. The aim of this review is to examine all the currently available strategies of pain management such as preemptive analgesia (PA), patient-controlled analgesia (PCA), and the various types of anesthesia that are used during the operation. With that objective in mind, we conducted our research by searching through the PubMed database for articles published in 2015 and after. For purely clinical reasons, we have attempted to classify all the best available evidence into three major categories: prior to surgery, during the surgery, and after the surgery. Multimodal analgesia seems to play a major role in the perioperative care of patients undergoing total hip arthroplasty (THA). Therefore, a considerable number of studies have been conducted analyzing all the current strategies that aim to minimize perioperative pain and consequent complications.

## Introduction and background

Osteoarthritis (OA) represents a major burden as it exerts a drastic impact on patients’ quality of life. It affects one in eight men and women in the US, and the final burden it causes is estimated to be between 1-2.5% of the gross domestic product (GDP) in developed countries [1]. The hip is one of the most commonly affected joints by OA. Total hip replacement (THR) constitutes an accepted and effective treatment modality for patients with end-stage hip OA. The primary indications for THR comprise cases refractory to conservative management and a substantial decrease in health-related quality of life [2]. As the population gets older, the number of THRs conducted is estimated to increase by twofold by 2030 compared to the numbers in 2005 [3]. However, this major surgical procedure is often accompanied by a great amount of postoperative pain leading to numerous adverse effects such as prolonged hospitalization and delayed rehabilitation, resulting in higher deep vein thrombosis rates [4]. Meticulous evaluation and management of pain are pivotal for the avoidance of the above-mentioned detrimental effects. 

Previous studies have described pain as the “fifth vital sign” and highlighted the amenable need for improved pain control [5]. To emphasize the important role of pain, a plethora of studies have been performed to determine the best available strategies for the management of pain. This article was intended to summarize the latest preoperative, intraoperative, and postoperative techniques currently used for pain relief in patients undergoing THR.

## Review

Materials and Methods

Terms Searched

We enagaged in a search of the following terms on PubMed in our quest to find articles related to pain management in patients undergoing THR: (1) “osteoarthritis” AND “hip”, (2) “total hip replacement” AND “pain management”, (3) “total hip arthroplasty” AND “pain management”, (4) “pain control” AND “total joint arthroplasty”, (5) “chronic pain management”, (6) “post-operative pain”, (7) “patient education”, (8) “arthroplasty” AND “rehabilitation”, (9) “multimodal analgesia”, (10) “preemptive analgesia” , (11) “nsaids” AND “pain management”, (12) “nsaids” AND “adverse effects”, (13) “opioids” AND “adverse effects”, (14) “gabapentin” AND “total hip replacement”, (15) “buprenorphine” AND “pain management”, (16) “anesthesia” AND “hip arthroplasty”, (17) “peri-articular injection” AND “hip arthroplasty”, (18) “pca” AND “ hip arthroplasty”, (19) “cryotherapy” AND “hip replacement”, and (20) “acetaminophen” AND “hip arthroplasty”.

Inclusion and Exclusion Criteria

Authors searched through PubMed and took into consideration articles published in 2015 and after. Evidence of level I-IV has been included in our review as there was no high-quality evidence for some topics. All the articles selected were in the English language, and we also excluded articles related to veterinary studies.

Data Extraction

The authors independently extracted the data. Titles, abstracts, and conclusions of each article were screened with the aim to identify any relevant articles. In addition, the reference list of each article was screened in order to find even more articles related to our topic.

Preoperative

I. Patient Education

Many studies have demonstrated the significance of preoperative education of patients undergoing THR. A review that was conducted in 2017 has recommended that every candidate for THA needs to be involved in discussions regarding their rehabilitation program as this process will help them develop a realistic perception regarding postoperative pain [6]. In this way, patients will be aware of what their expectations should be, which in turn can affect the length of hospital stay and the whole discharge planning. Furthermore, two more recent studies have highlighted the importance of education coupled with exercise prior to THR; the studies showed that education and exercise had a positive impact on postoperative pain, mobility function, and hospital length of stay [7,8]. On the other hand, an observational study involving 30,756 patients has demonstrated that preoperative patient education does not affect the amount of pain after the surgery. We feel there should be more randomized controlled trials (RCTs) on the subject as the current data provides an unclear and uncertain picture [9].

II. Preemptive Administration

Preemptive analgesia (PA) is a step that requires to be initiated prior to the surgical procedure when a painful stimulus is actually produced; however, it often needs to be stopped weeks before the surgery due to secondary risks of bleeding and delays in postsurgical healing. The main idea is to minimize the central sensitization and thereby to reduce the amount of pain postoperatively [10]. In addition, PA causes an increase in pain threshold, and therefore patients who undergo THA with prior PA tend to experience less chronic pain conditions after the surgery [11]. Some of the most commonly used PA medications are cyclooxygenase-2 (COX-2) inhibitors, nonsteroidal anti-inflammatory drugs (NSAIDs), opioids, and gabapentinoids [11,12].

COX-2 inhibitors: COX-2 inhibitors such as celecoxib and parecoxib represent a subtype of NSAIDs, capable of blocking the release of inflammatory cells such as prostaglandin E2 (PGE2) without having any detrimental effects on the epithelium of the stomach. The above-mentioned medications have been widely used for the treatment of acute pain [13]. A recent meta-analysis that collected data from 17 RCTs has demonstrated that patients who received COX-2 not only experienced less pain postoperatively but they also required a smaller quantity of opioids, which in turn reduced the rates of opioid-related side effects [14]. Similarly, according to a randomized, double-blind placebo-controlled trial, the administration of parecoxib to patients undergoing THA decreases the amount of postoperative pain and opioid consumption as well as the length of stay in the hospital [15]. Despite the fact that COX-2 can be administered without even increasing the risk of perioperative bleeding, it is worth mentioning that these medications are associated with a high risk of cardiovascular diseases [11,15].

NSAIDs: NSAIDs represent a class of medicines that have been widely used for their analgesic and anti-inflammatory effects, especially in surgical patients. This class of drugs leads to a decrease in prostaglandin production by the inhibition of the enzyme cyclooxygenase. A study conducted in 2016 has demonstrated that NSAIDs not only result in less postoperative pain but also a reduction in opioid consumption [16]. On the other hand, clinicians should administer these drugs with strict adherence to protocols as they may lead to adverse effects related to the gastrointestinal tract and cardiovascular system [17].

Opioids: opioids have always played a major role in the treatment of perioperative pain even though they may result in numerous side effects such as constipation, nausea, and respiratory depression in higher doses [18]. A survey that was conducted among the members of the American Association of Hip and Knee Surgeons has demonstrated that oxycodone, an opioid, is one of the most commonly used drugs as part of PA [19]. However, findings from another retrospective study have shown that the administration of oxycodone as part of PA can lead to an increase in postoperative opioid consumption coupled with greater postoperative pain [20]. Undoubtedly, further quality studies need to be conducted in order to determine the benefits of the preoperative use of opioids.

Gabapentinoids: gabapentin and pregabalin are the two main types of gabapentinoids. A handful of studies have demonstrated their positive effects when used as part of PA. A recent meta-analysis that was published in 2016 showed that gabapentin, which affects the nociceptive process, reduced the rates of postoperative opioid consumption in major orthopedic surgeries [21]. Additionally, another meta-analysis has suggested that preoperative administration of gabapentinoids is associated with less pain after the surgery and that pregabalin seems to be more efficient than gabapentin when administered orally [22].

III. Preoperative Application of Buprenorphine Transdermal Patch

Buprenorphine is an opioid receptor-partial agonist, and hence it can have both agonist and antagonist properties. Despite the fact that transdermal buprenorphine patch (TBP) is usually prescribed for the management of chronic pain, only a handful of studies have been conducted regarding its preoperative administration as part of PA. A recent prospective study including 50 patients undergoing total joint arthroplasty has reported that the use of a 10-mg TBP dose reduced the postoperative pain, which in turn reduced the number of postoperative analgesics consumed [23]. On the other hand, a different retrospective study showed that patients who used uninterrupted TBP preoperatively required more opioid analgesics after the surgery; however, this study had many limitations, including a lack of documentation regarding postoperative pain scores [24]. More studies are definitely required to fully determine the efficacy of TBPs.

Intraoperative

I. Types of Anesthesia

THR can be carried out either with general anesthesia (GA) or with regional anesthesia (RA), which can be further divided into spinal anesthesia (SA), epidural anesthesia (EA), and peripheral nerve block. Even though THR used to be performed mainly under GA, recent trends suggest that SAs and EAs have become widely popular in this surgical field [6]. This could be attributed not only to the considerable number of complications that may arise from GA but also to the better outcomes that RA has shown [11,25]. Also, a recent study conducted in 2017 has suggested that both SA and EA are associated with less postoperative pain and reduced need for analgesics [26]. On the other hand, a systematic review has indicated that even though neuraxial anesthesia (SA and EA) seems to be more effective, further studies are needed to prove its efficacy [27]. It is also worth mentioning that although peripheral nerve block (such as fascia iliaca or quadratus lumborum block) seems to be as efficient as the EA with even fewer side effects, the most frequently used types of anesthesia are either a single-shot SA or a combination of two or more different types [4]. To conclude, the vast majority of current evidence suggests that neuraxial anesthesia is generally considered to be more efficient than GA [28].

II. Intraoperative Periarticular Injection

Local infiltration analgesia (LIA) is a popular method that has been studied extensively. The main idea is to infuse a mixture of a long-acting, diluted local anesthetic agent with anesthetic adjuvants such as NSAIDs, COX-2 inhibitors, and ketamine. Some of the most commonly used local anesthetics are ropivacaine, traditional bupivacaine, and liposomal bupivacaine. It is worth mentioning that liposomal bupivacaine has been associated with more superior outcomes in patients undergoing THR compared to traditional bupivacaine [29]. Apart from that, a considerable number of studies have reported that LIA is associated with more sufficient postoperative analgesia and less opioid consumption [30-34]. Furthermore, another recent study has suggested that LIA not only represents an effective method of analgesia but it also leads to a reduced length of stay in the hospital [35].

Postoperative

I. Patient-Controlled Analgesia (PCA)

Patient-controlled analgesia (PCA) has been widely used to manage postoperative pain in patients undergoing THA. This noninvasive and easy-to-use analgesia involves a method that takes into account the baseline pain of patients and varies based on each patient’s physical traits. In PCA, opioids are often administered via a controlled infusion pump, and, therefore, PCA can result in severe side effects such as respiratory depression, nausea, and vomiting [10]. Even though different routes of PCA administration have been established, the intravenous route seems to be the most commonly used one [4]. A recent prospective randomized trial has highlighted the great efficacy of a PCA oral device in assuring less pain for patients following THR [36]. A network meta-analysis conducted in 2018 showed that the most superior outcomes relating to pain management were achieved when PCA was administered starting from 6-12 hours after THA [37].

II. Cryotherapy

Cryotherapy constitutes a method in which ice packs or cooled water are applied over the surgical site to offer pain relief. The main idea is to reduce the periarticular temperature by cooling the surrounding soft tissues and thereby minimizing both the topical blood flow and the nerve signal transmission. As a result, the inflammation process is interrupted. Numerous studies have demonstrated that cryotherapy can decrease postoperative pain. A systematic review has shown that cryotherapy specifically reduced the pain during the second postoperative phase of pain apart from ensuring considerably less loss of blood [38]. Additionally, a prospective cohort study has highlighted that patients who receive cryotherapy not only experience faster rehabilitation but they also spend less time at the hospital [39].

III. Acetaminophen

Even though the way in which acetaminophen acts has not been fully understood, numerous studies have demonstrated both its antipyretic and analgesic benefits. A recent randomized controlled trial suggested that the administration of acetaminophen as part of multimodal analgesia resulted in less postoperative pain coupled with reduced opioid consumption [40]. It is worth mentioning that another randomized study reported that there is no difference in pain scores between oral and intravenous administration of acetaminophen after the surgery [41]. On the contrary, another meta-analysis showed that perioperative use of acetaminophen did not affect pain scores after the surgery, yet its findings seem to be contradictory as the consumption of opioids reportedly decreased significantly in the subjects [42].

## Conclusions

THA is one of the most commonly performed surgical procedures globally even though it is often accompanied by a great amount of pain, especially during the first days following the surgery. Multimodal analgesia seems to offer efficient pain relief as its main concept involves using a variety of pain management tools that aim at different steps of the pain cascade. However, further studies involving not only orthopedic surgeons but also anesthetists and rehabilitation experts need to be conducted in order to definitively evaluate the benefits of the method.

## References

[1] O’Neill TW, McCabe PS, McBeth J (2018). Update on the epidemiology, risk factors and disease outcomes of osteoarthritis. Best Pract Res Clin Rheumatol.

[2] Aresti N, Kassam J, Nicholas N, Achan P (2016). Hip osteoarthritis. BMJ.

[3] Büttner M, Mayer AM, Büchler B, Betz U, Drees P, Susanne S (2020). Economic analyses of fast-track total hip and knee arthroplasty: a systematic review. Eur J Orthop Surg Traumatol.

[4] Min BW, Kim Y, Cho HM (2016). Perioperative pain management in total hip arthroplasty: Korean Hip Society guidelines. Hip Pelvis.

[5] Tompkins DA, Hobelmann JG, Compton P (2017). Providing chronic pain management in the ‘Fifth Vital Sign’ era: historical and treatment perspectives on a modern-day medical dilemma. Drug Alcohol Depend.

[6] Gaffney CJ, Pelt CE, Gililland JM, Peters CL (2017). Perioperative pain management in hip and knee arthroplasty. Orthop Clin North Am.

[7] Moyer R, Ikert K, Long K, Marsh J (2017). The value of preoperative exercise and education for patients undergoing total hip and knee arthroplasty: a systematic review and meta-analysis. JBJS Rev.

[8] Jönsson T, Ekvall Hansson E, Thorstensson CA, Eek F, Bergman P, Dahlberg LE (2018). The effect of education and supervised exercise on physical activity, pain, quality of life and self-efficacy - an intervention study with a reference group. BMC Musculoskelet Disord.

[9] Torisho C, Mohaddes M, Gustafsson K, Rolfson O (2019). Minor influence of patient education and physiotherapy interventions before total hip replacement on patient-reported outcomes: an observational study of 30,756 patients in the Swedish Hip Arthroplasty Register. Acta Orthop.

[10] Li JW, Ma YS, Xiao LK (2019). Postoperative pain management in total knee arthroplasty. Orthop Surg.

[11] Russo MW, Parks NL, Hamilton WG (2017). Perioperative pain management and anesthesia: a critical component to rapid recovery total joint arthroplasty. Orthop Clin North Am.

[12] Golladay GJ, Balch KR, Dalury DF, Satpathy J, Jiranek WA (2017). Oral multimodal analgesia for total joint arthroplasty. J Arthroplasty.

[13] Curtis E, Fuggle N, Shaw S (2019). Safety of cyclooxygenase-2 inhibitors in osteoarthritis: outcomes of a systematic review and meta-analysis. Drugs Aging.

[14] Jiang M, Deng H, Chen X (2020). The efficacy and safety of selective COX-2 inhibitors for postoperative pain management in patients after total knee/hip arthroplasty: a meta-analysis. J Orthop Surg Res.

[15] Xiao K, Yu L, Xiao W, Peng H, Bian Y, Wu Z, Weng X (2019). Pain management using perioperative administration of parecoxib for total hip arthroplasty: a randomized, double-blind, placebo-controlled trial. Pain Physician.

[16] Gupta A, Bah M (2016). NSAIDs in the treatment of postoperative pain. Curr Pain Headache Rep.

[17] Cooper C, Chapurlat R, Al-Daghri N (2019). Safety of oral non-selective non-steroidal anti-inflammatory drugs in osteoarthritis: what does the literature say?. Drugs Aging.

[18] Khademi H, Kamangar F, Brennan P, Malekzadeh R (2016). Opioid therapy and its side effects: a review. Arch Iran Med.

[19] Hannon CP, Keating TC, Lange JK, Ricciardi BF, Waddell BS, Della Valle CJ (2019). Anesthesia and analgesia practices in total joint arthroplasty: a survey of the American Association of Hip and Knee Surgeons membership. J Arthroplasty.

[20] Cooper HJ, Lakra A, Maniker RB, Hickernell TR, Shah RP, Geller JA (2019). Preemptive analgesia with oxycodone is associated with more pain following total joint arthroplasty. J Arthroplasty.

[21] Arumugam S, Lau CS, Chamberlain RS (2016). Use of preoperative gabapentin significantly reduces postoperative opioid consumption: a meta-analysis. J Pain Res.

[22] Mao Y, Wu L, Ding W (2016). The efficacy of preoperative administration of gabapentin/pregabalin in improving pain after total hip arthroplasty: a meta-analysis. BMC Musculoskelet Disord.

[23] Yadav M, Mohan CL, Srikanth I, Raj ER, Gopinath R, Chandrasekhar P (2019). Effect of preoperative application of buprenorphine transdermal patch on analgesic requirement in postoperative period in hip and knee replacement surgeries. J Anaesthesiol Clin Pharmacol.

[24] Martin YN, Pearson ACS, Tranchida JR, Weingarten TN, Schulte PJ, Sprung J (2019). Implications of uninterrupted preoperative transdermal buprenorphine use on postoperative pain management. Reg Anesth Pain Med.

[25] Wainwright TW, Gill M, McDonald DA (2020). Consensus statement for perioperative care in total hip replacement and total knee replacement surgery: Enhanced Recovery After Surgery (ERAS®) Society recommendations. Acta Orthop.

[26] Liang C, Wei J, Cai X, Lin W, Fan Y, Yang F (2017). Efficacy and safety of 3 different anesthesia techniques used in total hip arthroplasty. Med Sci Monit.

[27] Johnson RL, Kopp SL, Burkle CM (2016). Neuraxial vs general anaesthesia for total hip and total knee arthroplasty: a systematic review of comparative effectiveness research. Br J Anaesth.

[28] Memtsoudis SG, Cozowicz C, Bekeris J (2019). Anaesthetic care of patients undergoing primary hip and knee arthroplasty: consensus recommendations from the International Consensus on Anaesthesia-Related Outcomes after Surgery group (ICAROS) based on a systematic review and meta-analysis. Br J Anaesth.

[29] Asche CV, Ren J, Kim M, Gordon K, McWhirter M, Kirkness CS, Baurer BT (2017). Local infiltration for postsurgical analgesia following total hip arthroplasty: a comparison of liposomal bupivacaine to traditional bupivacaine. Curr Med Res Opin.

[30] Ma HH, Chou TA, Tsai SW, Chen CF, Wu PK, Chen WM (2019). The efficacy of intraoperative periarticular injection in total hip arthroplasty: a systematic review and meta-analysis. BMC Musculoskelet Disord.

[31] Bautista M, Muskus M, Llinás A, Bonilla G, Guerrero C, Moyano J (2018). Peri-articular injection of an analgesic mixture in primary total hip arthroplasty: an effective strategy for pain control during the first post-operative day. Int Orthop.

[32] Fusco P, Cofini V, Petrucci E (2018). Continuous wound infusion and local infiltration analgesia for postoperative pain and rehabilitation after total hip arthroplasty. Minerva Anestesiol.

[33] Titman S, Hommel A, Dobrydnjov I, Johansson A (2018). The efficacy of high volume of local infiltration analgesia for postoperative pain relief after total hip arthroplasty under general anaesthesia - a randomised controlled trial. Int J Orthop Trauma Nurs.

[34] Zhang X, Yang Q, Zhang Z (2017). The efficiency and safety of local liposomal bupivacaine infiltration for pain control in total hip arthroplasty: a systematic review and meta-analysis. Medicine (Baltimore).

[35] Nassar I, Fahey J, Mitchell D (2020). Rapid recovery following hip and knee arthroplasty using local infiltration analgesia: length of stay, rehabilitation protocol and cost savings. ANZ J Surg.

[36] Pizzi LJ, Bates M, Chelly JE, Goodrich CJ (2020). A prospective randomized trial of an oral patient-controlled analgesia device versus usual care following total hip arthroplasty. Orthop Nurs.

[37] Liu P, Wu Y, Liang Z, Deng Y, Meng Q (2019). Comparing the efficacy of pain managements after total hip arthroplasty: a network meta-analysis. J Cell Biochem.

[38] Ni SH, Jiang WT, Guo L, Jin YH, Jiang TL, Zhao Y, Zhao J (2015). Cryotherapy on postoperative rehabilitation of joint arthroplasty. Knee Surg Sports Traumatol Arthrosc.

[39] Okoro T, Ibrahim Y, Mansour N, Alderman P, Evans A (2019). The use of cryotherapy in the early postoperative period after total hip arthroplasty. Ortop Traumatol Rehabil.

[40] Takeda Y, Fukunishi S, Nishio S, Yoshiya S, Hashimoto K, Simura Y (2019). Evaluating the effect of intravenous acetaminophen in multimodal analgesia after total hip arthroplasty: a randomized controlled trial. J Arthroplasty.

[41] Westrich GH, Birch GA, Muskat AR (2019). Intravenous vs oral acetaminophen as a component of multimodal analgesia after total hip arthroplasty: a randomized, blinded trial. J Arthroplasty.

[42] Guo H, Wang C, He Y (2018). A meta-analysis evaluates the efficacy of intravenous acetaminophen for pain management in knee or hip arthroplasty. J Orthop Sci.

